# Estimating Coastal Winds by Assimilating High-Frequency Radar Spectrum Data in SWAN

**DOI:** 10.3390/s21237811

**Published:** 2021-11-24

**Authors:** Philip Muscarella, Kelsey Brunner, David Walker

**Affiliations:** Ocean Modeling Lab, SRI International, Ann Arbor, MI 48105, USA; kelsey.brunner@sri.com (K.B.); david.walker@sri.com (D.W.)

**Keywords:** high-frequency radar, waves, coastal winds, SWAN

## Abstract

Many activities require accurate wind and wave forecasts in the coastal ocean. The assimilation of fixed buoy observations into spectral wave models such as SWAN (Simulating Waves Nearshore) can provide improved estimates of wave forecasts fields. High-frequency (HF) radar observations provide a spatially expansive dataset in the coastal ocean for assimilation into wave models. A forward model for the HF Doppler spectrum based on first- and second-order Bragg scattering was developed to assimilate the HF radar wave observations into SWAN. This model uses the spatially varying wave spectra computed using the SWAN model, forecast currents from the Navy Coastal Ocean Model (NCOM), and system parameters from the HF radar sites to predict time-varying range-Doppler maps. Using an adjoint of the HF radar model, the error between these predictions and the corresponding HF Doppler spectrum observations can be translated into effective wave-spectrum errors for assimilation in the SWAN model for use in correcting the wind forcing in SWAN. The initial testing and validation of this system have been conducted using data from ten HF radar sites along the Southern California Bight during the CASPER-West experiment in October 2017. The improved winds compare positively to independent observation data, demonstrating that this algorithm can be utilized to fill an observational gap in the coastal ocean for winds and waves.

## 1. Introduction

Regional coastal modeling of the ocean and the atmosphere have come a long way in the last 20 years but most models still suffer from errors due to parameterization or inaccurate model inputs (i.e., bathymetry, initial or boundary conditions). These model errors can be addressed with the assimilation of local observations. In this study, we examine coastal wind estimation by assimilating observations of Doppler spectra from coastal high-frequency (HF) radar sites into a regional wave model with a variational assimilation approach. The wave model used here is SWAN ([1,2]) which is a state-of-the-art third-generation wave model used predominantly in coastal and inland waters.

HF radar data assimilation studies commonly utilize the surface current measurements traditionally associated with this data source. A few of these studies are [3,4,5]. The use of HF radar Doppler spectra to correct wave models is less widely used. Siddons et al. (2009) [6] examined the use of three different approaches for HF data assimilation into SWAN: (1) Ensemble Kalman Filter, (2) Ensemble Optimal Interpolation and (3) 3D variational scheme. This study makes use of beam-forming HF radars. Waters et al. (2013) [7] implemented an optimal interpolation assimilation approach that makes use of the windsea and swell parts of the spectrum separately. To the authors’ knowledge, the 4D-variational approach presented here has never been applied to HF radar data for wave modeling.

The methodology for the variational assimilation framework in the SWAN model was first laid out by Walker (2006) [8] and then used by Veeramony et al. (2010) [9]. The approach used only examined the spatial and spectral advection terms while ignoring source terms. Orzech et al. (2013, 2016) [10,11] expanded the implementation to include all the source terms with a new version of the adjoint of the wave-action-balance equations. Walker and Brunner (2021) [12] applied a stationary buoy assimilation setup with a similar methodology as presented here and showed good model skill when compared to both assimilated and unassimilated buoy observations.

Shore-based HF radars are routinely used to map surface currents by measuring the Doppler shifted backscatter from ocean waves. The backscattered energy from the ocean surface is due to Bragg scattering of the transmitted electromagnetic wave by ocean surface waves. Coherent reflections in the backscattered spectrum are generated at ocean wavelengths exactly $\frac{1}{2}$ the wavelength of the transmitted wave. Most applications make use of HF radars as a tool for producing high-resolution maps of surface currents. It is also possible to extract wave information from the second-order portion of the spectrum, where reflections are generated from waves at all frequencies not just the Bragg peaks. See [13] for a brief introduction to HFR theory.

The objectives of this work were to develop a system for assimilation of HF radar data for general nearshore domains, to produce improved wave nowcasts/forecasts, and provide wind forcing corrections. Initial implementation of our HF radar assimilation algorithm in SRI’s swanX 5DVAR system (based on SWAN 41.20) is complete. It includes a ground-wave HF radar model, based on first- and second-order Bragg scattering. The model uses the wave spectrum output from SWAN and the winds and ocean current field from COAMPS-OS along with the HF system parameters. The adjoint of the HF radar model translates errors in the HF radar spectrum into errors in the wave spectrum. The adjoint of SWAN then produces an effective wind error, consistent with the ST6 wind growth model. The whole system is converged, and wind field updates are made to the COAMPS wind inputs. Here, we present an initial demonstration of the SWAN HF radar assimilation system for CASPER-West experiment. Comparisons between the wind estimates from the SWAN HF assimilation and independent buoy data have shown the algorithm capable of correcting winds inputs for this coastal domain.

The rest of the paper has the following structure: Section 2 describes the SWAN and HFR forward scattering models; Section 3 presents the data assimilation framework including the cost function, adjoint models and implementation. The results of a real-world application of the assimilation are discussed in Section 4 followed by conclusions in Section 5.

## 2. The Models

The algorithm used here is based on the open-source SWAN wave spectrum model and makes use of HFR Doppler spectra data. We briefly describe the model, and then discuss the observed HFR Doppler spectra data. Then, we discuss the HFR forward model and the assimilation framework. Then, we examine the wind model and finish with a brief discussion about implementation.

### 2.1. SWAN Wave Model

SWAN is a third-generation wave model for coastal regions [1,14]. The basic equation used is the spectral balance equation for wave action N(x,s,t)=E(x,s,t)/σ, where *E* is the variance density spectrum, σ is the intrinsic wave radian frequency, x=(x,y) spatial location, s=(σ,θ) spectral location, and *t* is time. The action balance equation is given by

(1) $$\frac{\partial N}{\partial t}+\tilde{\nabla }\cdot \left(\tilde{C}N\right)=\frac{S_{tot}}{\sigma },$$

where $\tilde{\nabla }=\left(\frac{\partial }{\partial x},\frac{\partial }{\partial y},\frac{\partial }{\partial \sigma },\frac{\partial }{\partial \theta }\right)$, and $\tilde{C}=\left(C_{x},C_{y},C_{\sigma },C_{\theta }\right)$ are the wave-energy propagation velocities in physical and spectral space, consisting of wave group velocities plus ocean currents for physical space and the effects of refraction and wave–current interaction for spectral space. These are described in detail in [1], as is the source term on the right-hand side of Equation ( 1). This source term includes models for the effects of wind growth and loss of energy due to white-capping, bottom friction, and depth-induced breaking, as well as energy transfer within the spectrum due to nonlinear wave–wave interactions (resonant triad and quartet interactions). These source-term models can be written collectively as

(2) $$S_{tot}=S_{tot}\left(E\right)=A+S_{E}E,$$

where *A*, the so-called “linear” wind-growth term, is independent of *E*, and all other terms are proportional to *E*, but with a collective coefficient $S_{E}$ that depends at least weakly on *E*.

This set of equations can be solved for the action spectrum for spatial region Ω subject to appropriate initial conditions on Ω and boundary conditions on ∂Ω. For portions of the wave spectrum with propagation velocities that carry energy into Ω, the “incident” wave spectrum E(x,s,t) on ∂Ω must be specified. The spectrum is taken as periodic in θ and the σ boundaries are located far above and below the energy-containing region of the spectrum, yielding E=0 at these boundaries. In addition to these boundary and initial conditions, complete specification of the mathematical problem requires that the winds and bathymetry, including any tidal offset, be prescribed for Ω.

SWAN version 41.20 (used here) has been updated from 40.85 to include dissipation of swell energy, non-breaking dissipation, optional observation-consistent wind input, and white-capping physics; the complete suite of improvements is known as ST6. There are two options for the implementation of non-breaking dissipation. The first uses the formulation by Young et al. (2013) [15], updated by Zieger et al. (2015) [16], and the second uses the formulation by Ardhuin et al. (2010) [17]. We utilize the latter, with the default values. The details of the ST6 physics can be found in Rogers et al. (2012) [18]. Notably, this formulation changes the standard Komen et al. (1984) [19] wind speed scaling to a similar scaling with a free parameter, i.e., $U=S_{ws}u*$. Other concurrent changes introduced include changes to the wind drag formula and wave growth term, described in the SWAN documentation [20].

### 2.2. HF Radar Scattering Model

We start with the following expression for the observed energy in the Doppler spectrum $\hat{\sigma }$, as a function of Doppler frequency −∞<ω<∞, and range r>0, from a cross-loop HF radar system as the sum of the first- and second-order Bragg scattering contributions.

$$\begin{array}{cc} \hat{\sigma }\left(\omega ,r\right) & ={\hat{\sigma }}_{1}\left(\omega ,r\right)+{\hat{\sigma }}_{2}\left(\omega ,r\right)\;. \end{array}$$

The first-order scattering is given by

(3) $$\begin{array}{cc} {\hat{\sigma }}_{1}\left(\omega ,r\right) & =64\pi k_{0}^{4}\int_{\phi }w_{n}\left(\phi \right)\sum_{m=\pm 1}\int_{k}E\left(x,k\right)\delta \left(k-mk_{b}\right)\delta \left(\omega -m\omega_{b}\right)dk\;d\phi \;, \end{array}$$

where (r,ϕ) is the spatial polar coordinate system, centered on the radar, and $k_{0}$ is the electromagnetic wavenumber. Here, S(x,k) is the variance spectral density (the wave spectrum, equivalent to E(**x**,**s**) at a fixed time), k is the hydrodynamic wave vector, $k_{b}=-2k_{0}\left(\sin\phi ,\cos\phi \right)$ is the wave vector associated with the Bragg waves propagating toward the radar (here, waves at $-k_{b}$ are propagating away from the radar) and direction is idicated by m=±1. The intrinsic radian wave frequency $\omega_{k}=\sqrt{gk\tanh kh}$, where k=|k| and h=h(x) is the water depth, and $\omega_{b}=\omega_{k}$ evaluated for $k=k_{b}$. The illumination patterns $w_{n}\left(\phi \right)$ for the loop antennas are

(4) $$\begin{array}{c} w_{1}=\cos^{2}\left(\phi -\phi_{0}\right)\;\;\mathrm{and}\;\;w_{2}=\sin^{2}\left(\phi -\phi_{0}\right)\;, \end{array}$$

where $\phi_{0}$ is a reference for the antenna orientation.

At each spatial location, there is a near-surface ocean current u(x) and this will impart a Doppler shift Δω=k·u. This shift can be applied as follows,

(5) $$\begin{array}{cc} \hat{\sigma }\left(\omega ,r\right) & =\int_{\tilde{\omega }}\hat{\sigma }\left(\tilde{\omega },r\right)\delta \left(\tilde{\omega }+k_{b}\cdot u-\omega \right)\;d\tilde{\omega }\;; \end{array}$$

here, ω is Doppler-shifted so that $\omega =\tilde{\omega }+k_{b}\cdot u$. Applying to Equation (5) and integrating in $\tilde{\omega }$ yields

(6) $$\begin{array}{c} \begin{array}{cc} {\hat{\sigma }}_{1}\left(\omega ,r\right)= & 64\pi k_{0}^{4}\int_{\phi_{1}}^{\phi_{2}}w_{n}\left(\phi \right)\\ \; & \times \sum_{m=\pm 1}\left[\left\{\int_{k}E\left(x,k\right)\delta \left(k-mk_{b}\right)dk\;\right\}\delta \left(\omega -k_{b}\cdot u-m\omega_{b}\right)\;\right]d\phi \;; \end{array} \end{array}$$

this is the final form of the first-order Bragg-scattering model, where n=1,2 defines the antenna.

For second-order scattering, the contribution to the Doppler spectrum, equivalent to Equation (3), is

(7) $$\begin{array}{c} \begin{array}{cc} {\hat{\sigma }}_{2}\left(\omega ,r\right)= & 64\pi k_{0}^{4}\int_{\phi }\int_{k}w_{n}\left(\phi \right)\\ \; & \times \sum_{m,m^{\prime }=\pm 1}\;\;{\left|\Gamma \left(\omega ,k,k^{\prime },x\right)\right|}^{2}E\left(x,mk\right)E\left(x,m^{\prime }k^{\prime }\right)\delta \left(\omega -m\omega_{k}-m^{\prime }\omega_{k^{\prime }}\right)dk\;d\phi \;, \end{array} \end{array}$$

where $k^{\prime }=-m^{\prime }k_{b}-k$, with $m^{\prime }=\pm 1$, is an additional term defining propagation direction of the Bragg waves, and Γ is a coupling coefficient

(8) $$\begin{array}{c} \begin{array}{cc} \Gamma (\omega ,k,k^{\prime },x)= & \frac{kk^{\prime }}{2}\left[\frac{\cos\left(\phi -\varphi \right)\cos\left(\phi -\varphi^{\prime }\right)-2\cos\left(\varphi -\varphi^{\prime }\right)}{\sqrt{kk^{\prime }\cos\left(\varphi -\varphi^{\prime }\right)}-k_{b}\Delta }\right]\\ \; & -\;\;\frac{i}{2g}\left[\omega_{k}^{2}+\omega_{k^{\prime }}^{2}-2\omega_{k}\omega_{k^{\prime }}\frac{\omega^{2}+\omega_{b}^{2}}{|\omega^{2}-\omega_{b}^{2}|}\sin^{2}\left(\frac{\varphi -\varphi^{\prime }}{2}\right)\right]\\ \; & -\;\;\frac{i\omega }{2g}\left[\frac{m\omega_{k}^{3}{\mathrm{csch}}^{2}kh+m^{\prime }\omega_{k^{\prime }}^{3}{\mathrm{csch}}^{2}k^{\prime }h}{\omega^{2}-\omega_{b}^{2}}\right]\;, \end{array} \end{array}$$

where Δ=0.011−0.012i for seawater, and (k,φ) are polar coordinates in the wave spectrum *k*–plane. It should be noted that the last term in Γ goes to zero for deep water (kh→∞).

Accounting for the Doppler shift associated with near-surface currents yields

(9) $$\begin{array}{c} \begin{array}{cc} {\hat{\sigma }}_{2}\left(\omega ,r\right) & =64\pi k_{0}^{4}\int_{\phi }\int_{k}w_{n}\left(\phi \right)\;\sum_{m,m^{\prime }=\pm 1}\;\;{\left|\Gamma \left(\omega -k_{b}\cdot u,k,k^{\prime },x\right)\right|}^{2}\\ \; & \;\;\;\;\;\;\;\;\;\;\;\;\;\;\;\;\times E\left(x,mk\right)E\left(x,m^{\prime }k^{\prime }\right)\delta \left(\omega -k_{b}\cdot u-m\omega_{k}-m^{\prime }\omega_{k^{\prime }}\right)dk\;d\phi \;. \end{array} \end{array}$$

## 3. The Assimilation Framework

Assimilation of HFR data is carried out in a variational framework based on the models described above. This requires definition of a cost function and one or more control variables that are adjusted to minimize the cost function. In this case, the cost function is a measure of the error between the predicted and observed HFR Doppler spectrum, and the control variable is the surface wind field used in the SWAN model. The adjustments to the wind field are guided by the solution of an adjoint version of the SWAN model with input from the adjoint HFR model which, in turn, has as its input the error in the present HFR prediction. The gradient of the cost function with respect to the wind, used to adjust the wind field, is computed from the adjoint wave action spectrum, using an expression derived from the wind-growth term in the SWAN model. In the sections below, we first describe the cost function, followed by the adjoint forms of the models, and then the expression for the gradient. We then describe the sequence of operations for the assimilation algorithm.

### 3.1. Cost Function

The Doppler spectrum at second order has two components that are summed to produce the complete spectrum. Since they are summed, we can treat them separately and then combine the results. First, we must examine our data. The model for the first- or second-order returns produces the noise-free Doppler spectral density in units of normalized radar cross section (square meters per square meter) per radian (i.e., the units are rad${}^{-1}$). The data *s*, on the other hand, are uncalibrated and contain noise, and can be represented as

(10) $$s=\frac{\sigma }{C_{n}}+e,$$

where $C_{n}$ is a fixed (for channel *n*) calibration constant and *e* is a fixed noise level. Rearranging yields

(11) $$\sigma =C_{n}\left(s-e\right).$$

In practice, *e* can be estimated from the extreme upper and and lower frequency bands where the signal energy is unlikely to appear; however, this can lead to negative spectral density values in regions where s≈e. For this reason, we will use

(12) $$\sigma =C_{n}\left|s-e\right|.$$

For the systems of interest, the calibration constant $C_{n}$ is unknown, so construction of a cost function based on the error variance such as

(13) $$J=\int_{r}\int_{\omega }{\left[\sigma -\hat{\sigma }\right]}^{2}d\omega dr=\int_{r}\int_{\omega }{\left[C_{n}\left(s-e\right)-\hat{\sigma }\right]}^{2}d\omega dr\;,$$

where $\hat{\sigma }$ is the predicted spectrum, is problematic. We could attempt to estimate $C_{n}$ from the data by requiring the total backscattered energy, or the peak backscatter, to be the same for the model and data, but those approaches have issues of their own. If we define our cost function using the derivative in frequency of the difference in the log of the predicted and observed spectral density, we obtain

(14) $$\begin{array}{c} \begin{array}{cc} J & =\int_{r}\int_{\omega }{\left[\frac{\partial }{\partial \omega }\left\{\log\hat{\sigma }-\log(C_{n}\left|s-e|\right)\right\}\right]}^{2}d\omega dr\\ & =\int_{r}\int_{\omega }{\left[\frac{\partial }{\partial \omega }\left\{\log\hat{\sigma }-\log\left|s-e\right|\right\}\right]}^{2}d\omega dr. \end{array} \end{array}$$

We will use this form, which is insensitive to the unknown calibration constant, going forward.

### 3.2. Adjoint Models

Here, we describe the adjoint models used in the algorithm. We begin with the adjoint form of the SWAN model, followed by the adjoint forms of the first- and second-order HFR backscatter models.

The adjoint form of the SWAN model, described in Veeramony et al., 2010 [9], Orzech et al., 2016 [11], and Walker and Brunner 2021 [12], is

(15) $$\begin{array}{cc} \frac{\partial A}{\partial t}-\tilde{C}\cdot \tilde{\nabla }A\; & =S_{E}A-S_{D}\;, \end{array}$$

where A(x,s) is the adjoint wave action spectrum, and $S_{D}$ is an ‘observation’ term, discussed below. The adjoint SWAN model is solved backward in time over the period of interest, subject to homogeneous boundary conditions.

The HFR spectrum model has first- and second-order parts; this will result in two observation terms in Equation (15), $S_{D}=S_{D1}+S_{D2}$. For $S_{D1}$, we set $\hat{\sigma }={\hat{\sigma }}_{1}$ in *J* to get $J_{1}$; taking the first variation with respect to *E* yields

(16) $$\delta_{E}J_{1}=\int_{r}\int_{\omega }\frac{\partial }{\partial \omega }\left\{\log{\hat{\sigma }}_{1}-\log\left|s-e\right|\right\}\frac{\partial }{\partial \omega }\left(\frac{\delta_{E}{\hat{\sigma }}_{1}}{{\hat{\sigma }}_{1}}\right)d\omega dr\;.$$

Integrating by parts yields

(17) $$\delta_{E}J_{1}=\int_{r}\int_{\omega }\frac{1}{{\hat{\sigma }}_{1}}\frac{\partial^{2}}{\partial \omega^{2}}\left\{\log{\hat{\sigma }}_{1}-\log\left|s-e\right|\right\}\delta_{E}{\hat{\sigma }}_{1}d\omega dr,$$

where

(18) $$\delta_{E}{\hat{\sigma }}_{1}=64\pi k_{0}^{4}\int_{\phi }w_{n}\left(\phi \right)\sum_{m=\pm 1}\left[\left\{\int_{k}\delta E\left(k,x\right)\delta \left(k-mk_{b}\right)dk\right\}\delta \left(\omega -k_{b}\cdot u-m\omega_{b}\right)\right]d\phi .$$

Note that in Equation ( 17), integration by parts produces an additional term evaluated at the upper and lower boundaries of ω, where the spectrum, and hence this term, will vanish. Then, combining Equations ( 17) and ( 18) and re-arranging the order of integration results in

(19) $$\begin{array}{c} \begin{array}{c} \delta_{E}J_{1}=-64\pi k_{0}^{4}\int_{k}\int_{x}\sum_{m=\pm 1}\int_{\omega }\frac{\omega_{n}\left(\phi \right)}{r{\hat{\sigma }}_{1}}\frac{\partial^{2}}{\partial \omega^{2}}\left\{\log{\hat{\sigma }}_{1}-\log\left|s-e\right|\right\}\\ \delta \left(\omega -k_{b}\cdot u-m\omega_{b}\right)d\omega \delta \left(k-mk_{b}\right)\;\delta E\;dxdk\;, \end{array} \end{array}$$

where we have combined the *r* and ϕ integrations into a single 2D spatial integration in x (i.e., dx/r=drdϕ). The resulting contribution to the adjoint SWAN observation term is a portion of the integrand in this expression multiplying δE,

(20) $$\begin{array}{c} \begin{array}{c} S_{D1}\left(x,k\right)=-64\pi k_{0}^{4}\frac{\omega_{n}\left(\phi \right)}{r}\sum_{m=\pm 1}[\frac{1}{{\hat{\sigma }}_{1}\left(\omega ,r\right)}\frac{\partial^{2}}{\partial \omega^{2}}\left\{\log{\hat{\sigma }}_{1}\left(\omega ,r\right)-\log\left|s\left(\omega ,r\right)-e\right|\right\}\\ \delta \left(k-mk_{b}\right){]}_{\omega =m\omega_{b}+k_{b}\cdot u}\prime \end{array} \end{array}$$

where we have integrated in ω, evaluating the integrand at the shifted Bragg frequencies $\left(\omega =\pm \omega_{b}+k_{b}\cdot u\right)$. The gradient term is assigned at the Bragg wave-vector locations in the wave spectral domain $k=\pm k_{b}$ and, in the spatial domain, at x=(r,ϕ). For use in the SWAN assimilation algorithm, $S_{d}\left(k,x\right)=\delta S\left(k,x\right)$.

We now compute the the second-order contribution to the observation term $S_{D2}$. We start by inserting the expression for the second-order Doppler spectrum into the cost function and then take the first variation with respect to *E* and proceed as above for $S_{D1}$. This yields

(21) $$\begin{array}{c} \begin{array}{c} S_{D2}\left(x,k\right)=-64\pi k_{0}^{4}\frac{\omega_{n}\left(\phi \right)}{r}\sum_{m^{\prime }=\pm 1}{\left[\frac{1}{{\hat{\sigma }}_{1}\left(\omega ,r\right)}\frac{\partial^{2}}{\partial \omega^{2}}\left\{\log{\hat{\sigma }}_{1}\left(\omega ,r\right)-\log\left|s\left(\omega ,r\right)-e\right|\right\}\right]}_{\omega =\omega_{d}}\\ \times 2|\Gamma \left(\omega_{d},k,k^{\prime },x\right){|}^{2}E\left(x,-k-m^{\prime }k_{b}\right) \end{array} \end{array}$$

where $k^{\prime }=-mk_{b}-k,\omega_{d}=k_{b}\cdot u+m\omega_{k}+m^{\prime }\omega_{k^{\prime }}$ and $m,m^{\prime }=\pm 1$ where, again, these are for approaching/receding waves (respective halves of the k plane).

In $S_{D1}$ and $S_{D2}$, the error in the Doppler spectrum at $\omega_{d}$ is mapped to a correction in the wave spectrum at k and the correction is scaled by the scattering coefficient Γ and, for the second-order contribution, wave energy spectral density at the resonant wavenumber.

The final piece needed for the assimilation algorithm is an expression for the gradient of the error in the Doppler spectrum (the cost function) with respect to the wind that can be used to guide adjustments to the wind. The source function *S* on the right-hand side of the SWAN model Equation (1) contains the wind-growth source term, $S_{in}$ (detailed in [14]). The desired gradient is obtained by taking the first variation with respect to the vector wind. The first variation of the cost function *J* with respect to the vector wind $U_{w}$ is

(22) $$\delta_{U_{w}}J=\int_{T}\int_{R}\left\{-\int_{S}\frac{1}{\sigma }\frac{\delta S_{in}}{\delta U_{w}}Ads\right\}\cdot \delta U_{w}dxdt,$$

where the vector nature of the wind is acknowledged. This equation governs the reduction in *J* that will result from a change in $U_{w}$. To reduce *J*, we set $\delta U_{w}$ the incremental change in the wind to

(23) $$\delta U_{w}\left(x,t\right)=\alpha \int_{S}\frac{1}{\sigma }\frac{\delta S_{in}}{\delta U_{w}}Ads,$$

where α is positive. In the SWAN model, there are many choices for $S_{in}$, and they all are somewhat complex empirical parameterizations. For this reason, we compute $\delta S_{in}/\delta U_{w}$ by perturbing the vector wind components by a small amount, and calculating the change in $S_{in}$, divided by the component perturbation. The result is integrated over the spectral domain to yield a spatially-resolved time-varying gradient to guide the correction to the vector wind components.

### 3.3. Implementation

The initial guess for the wave spectrum over the entire region is a previously corrected SWAN model background that has corrected the swell due to assimilation of National Data Buoy Center (NDBC) buoy data. The adjoint Equation (15) is solved using the HF radar observations as input, the gradient. The gradient is then used in a conjugate-gradient procedure [21] to calculate the wind field that results in the minimum of the cost function *J*. In practice, the following sequence of steps is followed:

- The adjoint solution is calculated using the error in the most recent prediction as input;
- Using the adjoint solution, the gradient is determined;
- The conjugate-gradient descent algorithm is used to calculate a new estimate of the wind field and $U_{10}$;
- The SWAN model is run with corrected inputs and a new wave-spectrum prediction for the region is generated;
- The forward HF radar model is run with the new spectrum as input and a new prediction of the data is generated.

**Figure 1:**
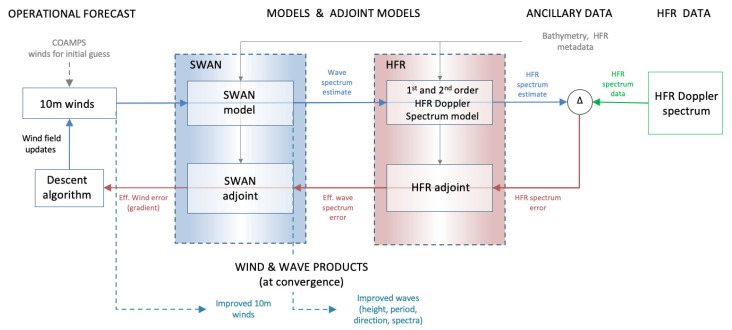
Algorithm flow chart. The algorithm makes use of operation forecast data (as a first guess for winds), models and adjoint models, ancillary data (such as bathymetry), and HF radar Doppler data. The algorithm outputs are wind and wave products, improved wave spectra and improved estimates of the wind field.

A graphical description of the complete algorithm structure is shown in Figure 1. The algorithm makes use of operation forecast data from the COAMPS model (as a first guess for the winds), models and adjoint models (the SWAN wave spectrum model and the HF radar forward spectrum model and corresponding adjoints), ancillary data such as bathymetry, and HF radar Doppler spectrum data (from CODAR HFR sites). The algorithm outputs are wind and wave products, improved wave spectra and improved estimates of the wind field. Estimates begin with a first guess wind field for the region obtained from operational wind forecasts. Estimates of the wave spectrum and the HF radar Doppler spectrum are calculated. The HF radar Doppler spectrum is compared to that for the data (from the CODAR sites) and the difference is fed back through the adjoint HFR and SWAN models. The gradient of the error in the estimated HFR spectrum with respect to the wind field is calculated from the adjoint wave spectrum. This gradient is used to adjust the wind field using a descent algorithm and the steps are repeated until the wind field converges and the HFR spectrum is a best fit.

## 4. Results

The HFR inverse modeling framework developed here was applied for estimation of the 10-m winds in the Southern California Bight during the CASPER-West field experiment in October 2017. The aim here was to utilize HF radar Doppler spectra observations to estimate the winds near the coast. The SWAN model (version 41.20) was run with all the relevant model physics enabled (i.e., wind generation, bottom friction, three-wave and four-wave interactions, depth-induced breaking, dissipation of swell energy (non-breaking dissipation) and optional observation-consistent wind input and white-capping physics). The complete suite of improvements are known as ST6 with all input parameters set to their default values. The details of the ST6 physics can be found in Rogers et al. [18]. The initial guess for each 24-h assimilation window was taken from a previously computed SWAN assimilation using NDBC buoy observations to correct the open boundary conditions. The algorithm product is a wave and wind estimate for the region from a non-stationary SWAN model run for the time of interest that is a best fit to the observational data.

### 4.1. Problem Setup

**Figure 2:**
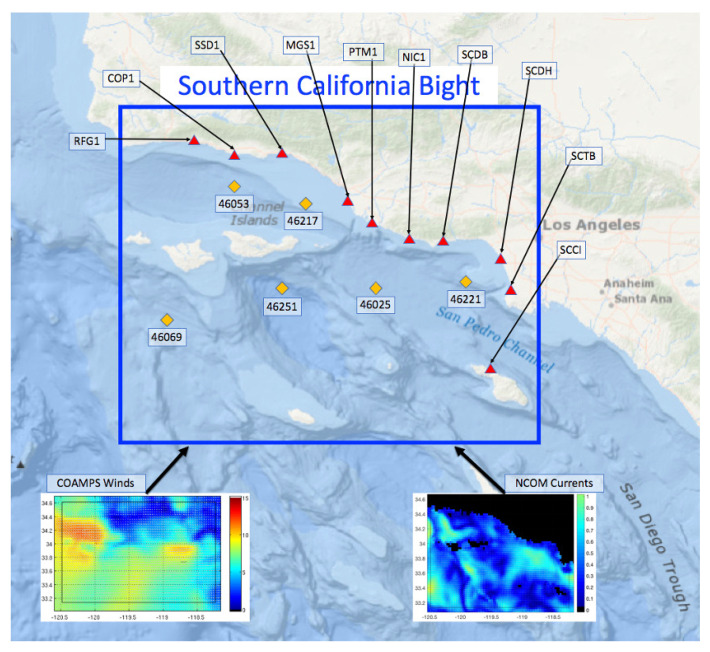
Map of the Southern California Bight with 10 HF radar sites (red triangles) and 6 NDBC buoys (orange diamonds). Example input fields for the SWAN wave model from COAMPS and NCOM are on the bottom.

Figure 2 shows the extent of the computational domain. The SWAN model domain was setup extending from 118.371${}^{\circ }$ E to 120.4822${}^{\circ }$ E (Δx = 0.033${}^{\circ }$) and 33.1376${}^{\circ }$ N to 34.6132${}^{\circ }$ N (Δ = 0.036${}^{\circ }$). The directional grid has 72 bins for a resolution of $\Delta \theta =5^{\circ }$ and the frequency grid has 49 bins, logarithmically spaced, covering the frequency range 0.05 Hz to 0.5 Hz. Wind forcing from 6-km COAMPS-OS and surface currents from 3-km NCOM are used as inputs to the wave model. Overlapping 24-h model runs are initialized every 12-h from hotstart files to cumulatively cover the length of the CASPER-West experiment from 1–23 October 2017. As mentioned previously, the assimilation model runs use wave directional spectrum parameters (a’s and b’s) from six directional wave buoys (46069, 46251, 46217, 46025, 46221, and 46222) within the model domain to apply a correction to the open boundary wave conditions.

### 4.2. HF Radar Data Description

Here, we utilize the Doppler spectra returns of sea state from 10 SeaSonde type Coastal Ocean Dynamics Applications Radar (CODAR). The locations of HFR sites can be seen in Figure 2. Due to the nature of the HF radar, observations are generated on a polar coordinate grid centered on the antenna locations. The range cells on this polar coordinate grid are spaced at 1.5 km. The Doppler spectra data from ocean waves are received at each HF site and are calculated by spectral processing of the signal within each range bin. The received signal was sampled at approximately 2 Hz and Fourier-transformed with a 512 sample window, resulting in a Doppler spectrum being recorded approximately every 4 min, each spectrum containing 512 Doppler frequency samples from −1 to +1 Hz. The data used for this study include these raw Doppler spectra, which are not published online, for the 10 CODAR antennas. Each site consists of two cross-loop antennas and one omnidirectional antenna. The omnidirectional data was not considered to be useful for this study. For purposes of comparison to model predictions, the data were averaged over 3 range cells and a roughly 1-h time interval. Thus, hourly predictions of the Doppler spectrum were made for 8 range samples from 10–42 km.

**Figure 3:**
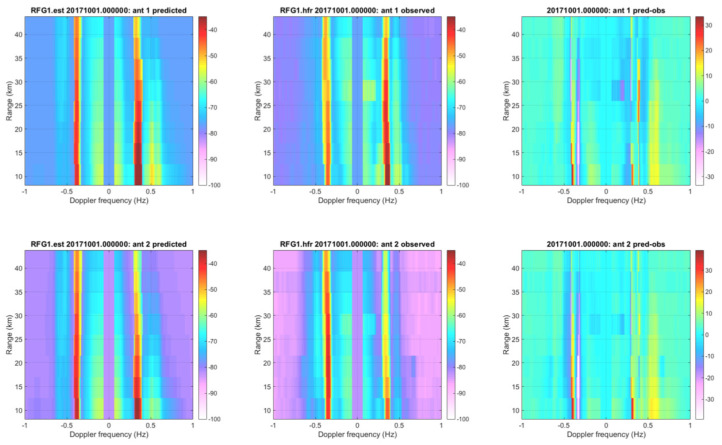
Range Doppler plots for the RFG1 HF radar site antennas 1 and 2 on 1 October 2017 at 0000z. The left plots are the predicted fields from the SWAN HF Doppler model. The center plots are the observed HF radar data. The right plots denote the difference between the first two fields. The first- and second-order Bragg peaks are visible in both the predicted and observed range Doppler plots.

Figure 3 shows an example comparison of range-Doppler plots for the Refugio State Beach (RFG1) HF radar site. The 1st order Bragg peaks are visible in both the observed and predicted data. The 1st order peak for the positive Doppler frequencies (waves coming towards the antenna) is stronger than for the negative Doppler frequencies (wave receding from the antenna). This trend is more prominent in the predicted spectra than the observed spectra although it is still present. The second-order peaks are also visible in both the predicted and observed plots although much less obvious than the first-order peaks.

### 4.3. Comparisons to Buoy Wave Data

In this section, we present comparisons between wave parameters from NDBC buoy 46217 and the predicted wave spectra from the same location. Comparison at buoys 46025 and 46221 are similar, so they are not shown in detail. We limit our comparisons to the significant wave height $H_{s}$, the mean wave period $T_{m}$, and the mean wave direction $\theta_{m}$. The figures presented here show the general performance of the algorithm at these buoy locations before we move on to the wind comparison.

**Figure 4:**
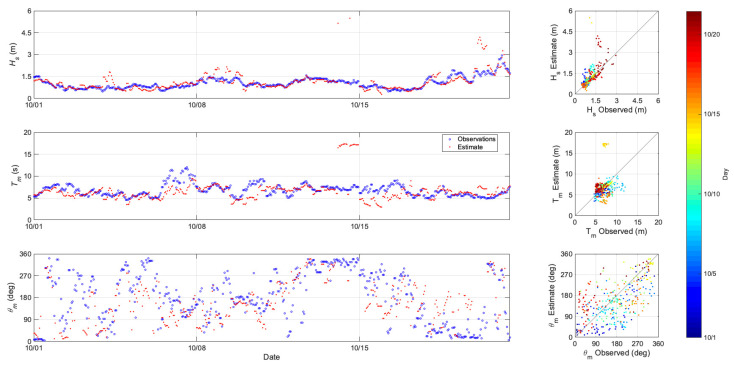
Comparison of estimated to observed wave parameters for buoy 46217. Top left panel is a time series of significant wave height $H_{s}$; the middle left panel is a time series of mean wave period $T_{m}$; and the bottom left panel is a time series of mean wave direction $\theta_{m}$. In the time series, the open circles show the observed quantities, the red dots show the HFR assimilation estimate. The right panels are the associated scatter plots colored by experiment day.

Figure 4 shows a comparison of the estimated wave parameters to the observed data from buoy 46217 in the Anacapa Passage near the Channel Islands. The water depth at this buoy location is 114 m. The top panel of Figure 4 is a time series of the estimated $H_{s}$, compared to the buoy observations. There is good agreement between the estimated and observed values for the majority of the study period. There are some times where the predicted $H_{s}$ overestimates the observations. This is most likely due to a poor specification of the boundary conditions during several assimilation cycles. The middle panel of Figure 4 shows the time series comparisons for $T_{m}$. Again, there is good agreement for a large portion of the month with the prediction underestimating the $T_{m}$ for several times. The bottom panel of Figure 4 is the time series comparison for $\theta_{m}$. The observations of $\theta_{m}$ show a wide spread of values with estimates generally following the same pattern as the observations. Quantitative error statistics for $H_{s}$, $T_{m}$, and $\theta_{m}$ for buoy 46217 are presented in Table 1.

### 4.4. Comparisons to Buoy Wind Data

Here, we compare the 10-m winds from the SWAN HF assimilation algorithm against winds from NDBC buoy 46053. The buoy winds have not been incorporated in any way into our assimilation framework, so, they can be treated as independent observations. We restrict our comparisons to the U and V components of the wind.

**Figure 5:**
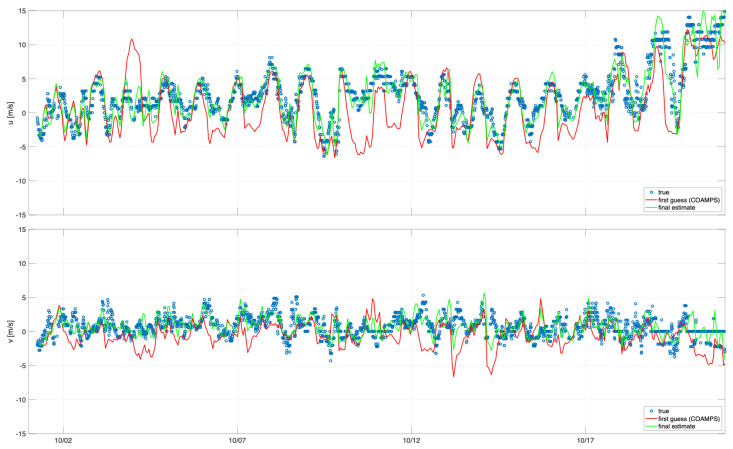
Wind comparison for U and V 10-m winds for NDBC buoy 46053 (blue circles), COAMPS first guess (red) and SWAN HF assimilation model (green) over the first 23 days of October 2017. In order to construct a continuous wind record from the 24-hour assimilation cycles, only the middle 12 h from each cycle were used.

**Figure 6:**
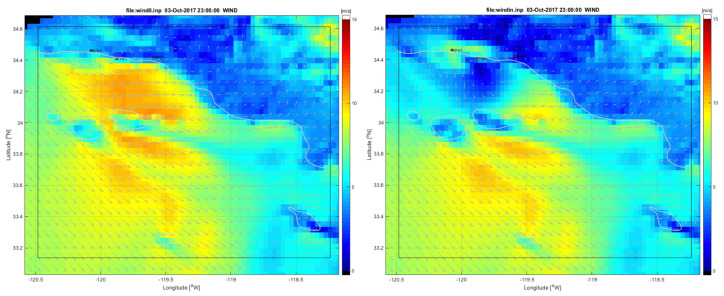
A 10-m wind field comparison for COAMPS first guess (left) and SWAN HF assimilation winds (right) for 3 October 2017 at 2300z. Notice the strong winds in the Santa Barbara Channel in the first guess that is not present in the assimilation estimate.

Figure 5 shows the comparisons for buoy 46053 located East of Santa Barbara in a depth of 405 m. This continuous record of 10-m winds was generated using the middle 12 h from each of the 24-hour assimilation cycles. The comparison shows that the SWAN wind estimate solution closely matches the winds from the NDBC buoy for the majority of this study period. The COAMPS first guess solution does not agree with the buoy data as well as the HF SWAN solution. Notably, in the U time series (top panel), there are several times on 3 October and 10 October where there is a significant difference between the first guess and the assimilation estimate where the assimilation estimate agrees with the observations. This points towards errors in the coastal wind field coming from COAMPS. Figure 6 shows a side-by-side field plot of the 10-m winds on 3 October 2017 at 2300 z. The left panel shows the COAMPS first guess winds that contain a strong eastward (positive U) wind feature in the Santa Barbara Channel. The right panel shows the SWAN HF assimilation 10-m winds with much weaker winds in the Santa Barbara Channel relative to the COAMPS first guess. The observations from buoy 46053 agree with the SWAN HF assimilation winds and show the potential for corrections to errors in coastal wind forecasts.

## 5. Discussion and Conclusions

In this work, we have described the development, implementation, and testing of an HF radar assimilation algorithm for the SWAN model. This framework utilizes a cost function to determine the error between the observed and predicted Doppler spectra from an HF radar first- and second-order forward model. Adjoint models of both the HF radar forward model and the SWAN model are then solved. The first- and second-order gradients of the cost functions are calculated from the adjoint spectrum solutions. Lastly, a conjugate-gradient algorithm is used to determine the appropriate Doppler spectrum and wind conditions that minimizes the cost function.

The algorithm was applied and validated using real-world observations from coastal wave buoys and HFR sites during the CASPER-West field experiment in October of 2017. The background wave field was taken from a buoy assimilation experiment where the open-boundaries were corrected. Predicted and observed significant wave height $H_{s}$, mean wave period $T_{m}$, and mean wave direction $\theta_{m}$ agreed well over the study period with a few short periods of disagreement.

In conclusion, a variational HF radar Doppler spectrum assimilation algorithm for the SWAN model has been developed. Corrections in the near surface winds during the CASPER-West experiment were generated. A comparison of these winds to independent buoy observations shows good agreement and the skill of the algorithm in adjusting errors in coastal wind forecasts. Additional testing of the algorithm in other regions of interest is necessary before this tool can be utilized for operational forecasting. Further expansion of the algorithm to potentially account for bistatic or skywave HF radar configurations is ongoing and should allow for more general utility and relocatablity.

## Figures and Tables

**Table 1:** Error statistics for significant wave height ($H_{s}$), mean wave period ($T_{m}$) and mean wave direction ($\theta_{m}$) from comparison of the SWAN assimilation results to observation data used in the background. The mean error in quantity *X* is ${\bar{\epsilon }}_{X}$, the RMS error is $\epsilon_{X}^{rms}$. For comparison, the mean and standard deviation of the observations ($\bar{X}$ and $\sigma_{X}$) are included on the second line, in parentheses.

Station	${\bar{\epsilon }}_{H_{s}}$	$\epsilon_{H_{s}}^{\mathrm{rms}}$	${\bar{\epsilon }}_{T_{m}}$	$\epsilon_{T_{m}}^{\mathrm{rms}}$	${\bar{\epsilon }}_{\theta_{m}}$	$\epsilon_{\theta_{m}}^{\mathrm{rms}}$
	(${\bar{H}}_{s}$)	($\sigma_{H_{s}}$)	(${\bar{T}}_{m}$)	($\sigma_{T_{m}}$)	(${\bar{\theta }}_{m}$)	($\sigma_{\theta_{m}}$)
46217	0.67 m	3.13 m	−0.16 s	2056 s	−8.67°	93.18°
	(1.05 m)	(0.40 m)	(6.77 s)	(1.36 s)	(180.80°)	(102.59°)

## Data Availability

All wave buoy and bathymetry used in this study are publicly available from the U.S. National Oceanic and Atmospheric Administration. The HFR Doppler spectra dataset is a level 0 data product that is not routinely reported, but can be obtained directly from the site operators.

## Abbreviations

The following abbreviations are used in this manuscript:

HFR	High-Frequency Radar
SWAN	Simulating WAves Nearshore
CODAR	Coastal Ocean Dynamics Applications Radar
NDBC	National Data Buoy Center
COAMPS	Coupled Ocean Atmosphere Modeling and Prediction System
NCOM	Navy Coastal Ocean Model

## References

[1] Booij N., Ris R.C., Holthuijsen L. (1999). A third-generation wave model for coastal regions: 1. Model description and validation. J. Geophys. Res. Ocean..

[2] Holthuijsen L. (2009). Waves in Oceanic and Coastal Waters.

[3] Paduan J., Shulman I. (2004). HF radar data assimilation in the Monterey Bay area. J. Geophys. Res..

[4] Ngodock H., Muscarella P., Carrier M., Souopgui I., Smith S. (2015). Assimilation of HF Radar Observations in the Chesapeake-Delaware Bay Region Using the Navy Coastal Ocean Model (NCOM) and the Four-Dimensional Variational (4DVAR) Method. Coastal Ocean Observing Systems.

[5] Marmain J., Molcard A., Forget P., Barth A., Ourmières Y. (2014). Assimilation of HF radar surface currents to optimize forcing in the northwestern Mediterranean Sea. Nonlinear Process. Geophys. (Eur. Geosci. Union).

[6] Siddons L., Wyatt L., Wolf J. (2009). Assimilation of HF radar data in the SWAN wave model. J. Mar. Syst..

[7] Waters J., Wyatt L., Wolf J., Hines A. (2013). Data Assimilation of partitioned HF radar wave data into Wavewatch III. Ocean Model..

[8] Walker D. (2006). Assimilation of SAR Imagery in a Nearshore Spectral Wave Model.

[9] Veeramony J., Walker D., Hsu L. (2010). A variational data assimilation system for nearshore application of SWAN. Ocean Model..

[10] Orzech M., Veeramony J., Ngodock H. (2013). A variational assimilation system for nearshore wave modeling. J. Atmos. Ocean. Technol..

[11] Orzech M., Veeramony J., Ngodock H., Flampouris S., Souopgui I. (2016). Recent Updates to SWANFAR, a 5DVAR Data Assimilation System for SWAN.

[12] Walker D., Brunner K. (2021). Estimating Nearshore Waves by Assimilating Buoy Directional Spectrum Data in SWAN. J. Ocean. Atmos. Technol..

[13] Paduan J., Graber H. (1997). Introduction to high-frequency radar: Reality and myth. Oceanography.

[14] Ris R.C., Holthuijsen L.H., Booij N. (1999). A third-generation wave model for coastal regions: 2. Verification. J. Geophys. Res. Ocean..

[15] Young I., Babanin A., Zieger S. (2013). The Decay Rate of Ocean Swell Observed by Altimeter. J. Phys. Oceanogr..

[16] Zieger S., Babanin A., Rogers W., Young I. (2015). Observation-based source terms in the third-generation wave model WAVEWATCH. Ocean Model..

[17] Ardhuin F., Rogers E., Babanin A.V., Filipot J.F., Magne R., Roland A., van der Westhuysen A., Queffeulou P., Lefevre J.M., Aouf L. (2010). Semiempirical Dissipation Source Functions for Ocean Waves. Part I: Definition, Calibration and Validation. J. Phys. Oceanogr..

[18] Rogers W., Babanin A., Wang D. (2012). Observation-consistent input and white-capping dissipation in a model for wind-generated surface wave: Description and simple calculations. J. Atmos. Ocean. Technol..

[19] Komen G.J., Hasselmann S., Hasselmann K. (1984). On the Existence of a Fully Developed Wind-sea Spectrum. J. Phys. Oceanogr..

[20] (2019). SWAN Scienctific and Technical Documentation.

[21] Polak E. (1971). Computational Methods in Optimization.

